# Application of iron oxide nanoparticles in the diagnosis and treatment of leukemia

**DOI:** 10.3389/fphar.2023.1177068

**Published:** 2023-03-30

**Authors:** Yiling Wang, Yan Yang, Xi Zheng, Jianyou Shi, Lei Zhong, Xingmei Duan, Yuxuan Zhu

**Affiliations:** Personalized Drug Therapy Key Laboratory of Sichuan Province, Sichuan Academy of Medical Science & Sichuan Provincial People’s Hospital, University of Electronic Science and Technology of China, Chengdu, China

**Keywords:** leukemia, iron oxide nanoparticles, magnetism, drug delivery, targeted therapy

## Abstract

Leukemia is a malignancy initiated by uncontrolled proliferation of hematopoietic stem cell from the B and T lineages, resulting in destruction of hematopoietic system. The conventional leukemia treatments induce severe toxic and a long series of unwanted side-effects which are caused by lack of specificity of anti-leukemic drugs. Recently, nanotechnology have shown tremendous application and clinical impact with respect to diagnosis and treatment of leukemia. According to considerable researches in the context of finding new nanotechnological platform, iron oxide nanoparticles have been gained increasing attention for the leukemia patients use. In this review, a short introduction of leukemia is described followed by the evaluation of the current approaches of iron oxide nanoparticles applied in the leukemia detection and treatment. The enormous advantages of iron oxide nanoparticles for leukemia have been discussed, which consist of the detection of magnetic resonance imaging (MRI) as efficient contrast agents, magnetic biosensors and targeted delivery of anti-leukemia drugs by coating different targeting moieties. In addition, this paper will briefly describe the application of iron oxide nanoparticles in the combined treatment of leukemia. Finally, the shortcomings of the current applications of iron-based nanoparticles in leukemia diagnosis and treatment will be discussed in particular.

## 1 Introduction

Leukemia is a tumor of the hematopoietic and lymphoid tissues. Clonal leukemia cells proliferate, accumulate and infiltrate in the bone marrow and other hematopoietic and non-hematopoietic tissues due to abnormal proliferation of differentiated hematopoietic stem cell or progenitor cells, thus affecting the physiological bone marrow of various tissues and organs. Siegel et al. (2019) Symptoms are usually characterized by bleeding, fever, immunosuppression, bone and joint pain, hepatosplenomegaly, and lymphadenopathy, these symptoms are caused by varying degrees of anemia, neutrophil, thrombocytopenia, or tissue infiltration. Chen and Zhou (2012) According to the progression of the disease, leukemia is mainly classified as acute and chronic, while leukemia is mainly classified as lymphoid and myeloid leukemia according to the origin of malignant proliferating cells. Alsalem et al. (2018) Current therapies of leukemia include chemotherapy, radiotherapy, targeted therapy and stem cell transplantation. Kadia et al. (2015) Although, many studies have shown that viruses, genetics, radiation and chemical toxins may cause leukemia, the cause of leukemia is still not a unified answer. Curtis et al. (1992); Lo et al. (2010); Winandy et al. (1995) The other hand, the clinical characteristics of different types of leukemia are obviously different. Because of the limitation of the above factors, there is no uniform treatment of leukemia at present. Short et al. (2018).

Chemotherapy is the most common treatment for leukemia. Pant and Bhatt (2017) Chemotherapy drugs such as vinblastine, Cytarabine, Vindesine, daunorubicin, northromycin, doxorubicin, and mitoxantrone are often used. Single drug often leads to drug resistance, so combination therapies are used to treat leukemia in order to reduce even eliminate drug resistance. Kantarjian et al. (2010) The “3 + 7 regimen” proposed in the 1970s has become the cornerstone of induction chemotherapy for acute myeloid leukemia (AML), which can achieve a certain remission rate. However, for older patients with leukemia, this regimen can lead to up to 30% early treatment-related mortality and the survival rate is less than 10 percent in 3–5 years Gardin et al. (2020).

Radiotherapy is the treatment of malignant tumors with radionuclide alpha, beta, gamma radiation and X-ray, electron and proton beams produced by various X-ray therapy machines or accelerators. Walter (2022) Radiation inevitably exposes normal cells to ionizing radiation, so there is a risk that cancer may develop again during treatment. Bodet-Milin et al. (2016).

Targeted therapy, as one of the treatment modalities for leukemia, has three main therapeutic mechanisms: small molecule inhibitors that target gene mutations, inhibitors that target key signaling pathways, and antibodies or antibody-coupled drugs that target cell surface molecules. Newell and Cook (2021) Although great progress has been made in the treatment of leukemia with targeted therapy, there are still many problems. For example, treatment of leukemia with FLT 3 inhibitors alone, such as Gilteritinib, is less toxic than conventional chemotherapy, but resistance continues to develop in patients with certain gene mutations. Travis et al. (2000).

Currently, Hematopoietic stem cell transplantation (HSCT) is considered to be an effective treatment for leukemia. Ljungman et al. (2019) HSCT is to kill as many abnormal cells or tumor cells as possible through high-dose chemotherapy or radiotherapy, and then restore hematopoietic and immune functions through intravenous infusion of pre-collected autologous or allogeneic stem cells. Pavlů and Apperley (2013) However, HSCT also cause some adverse reactions. After allogeneic stem cell transplantation, the need for immunosuppressants, may cause low immunity, secondary infection. Zhao et al. (2019) Moreover, some patients may have a graft-versus-host reaction, which not only leads to graft failure, but also to death. Peccatori and Ciceri (2010).

The above-mentioned therapies generally have some disadvantages, such as low drug targeting, high adverse reactions, and drug resistance in patients. Therefore, it is very important to develop an efficient and targeted intelligent drug delivery system in the past decades Wicki et al. (2015) With the continuous progress of nanotechnology, nanotechnology has been widely used in drug delivery, disease diagnosis and treatment (Patra et al., 2018).

Iron oxide nanoparticles (IONPs) in the diagnosis and treatment of cancer has attracted more and more attention. Wang et al. (2018) Furthermore, iron-based nanoparticles have high transverse relaxation rate, good biocompatibility and long circulation *in vivo*, has been widely used as magnetic resonance imaging (MRI) contrast agent for tumor diagnosis. Na et al. (2009); Lee et al. (2012) Iron oxide nanoparticles have unique magnetic properties and can be used as magnetic thermotherapy, photothermal therapy and magnetic targeted drug delivery. Alphandéry (2019); Li et al. (2017) Based on its characteristics, it has been used more and more in hematological tumors in recent years.

**FIGURE 1:**
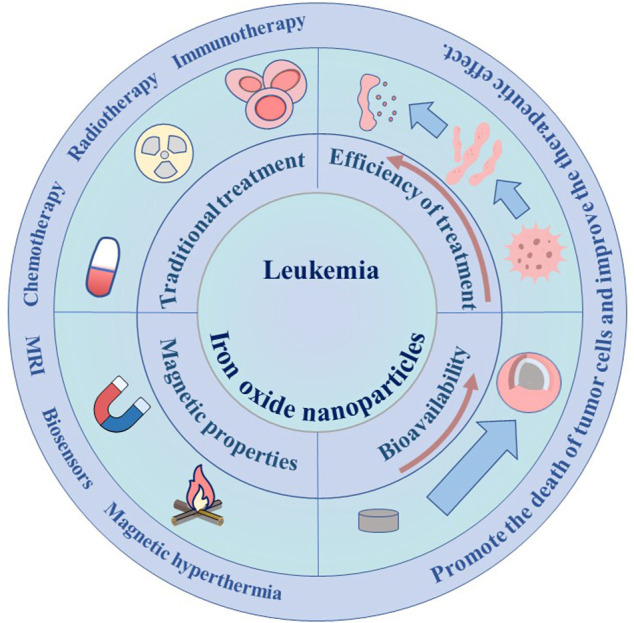
Application of IONPs in leukemia.

In this paper, the current status of leukemia treatment, the preparation of iron nanoparticles and their application in leukemia detection and treatment are briefly introduced (Figure 1). This article reviews the great advantages of iron nanoparticles in the treatment of leukemia, including MRI detection as an effective contrast agent, tumor-related iron-based nanoparticle therapy, and targeted drug delivery by coating different targets. In addition, this paper will briefly introduce the application of iron-based nanoparticles in combination therapy of leukemia. Finally, the shortcomings of the current application of iron-based nanoparticles in the diagnosis and treatment of leukemia will be particularly discussed.

## 2 Iron-based nanoparticles

In recent years, nanotechnology has been widely used in the diagnosis and treatment of various diseases, especially in oncology. El-Sherbiny et al. (2017) Nanomedicine overcomes many disadvantages of traditional cancer treatment methods for cancer treatment, such as low bioavailability, drug resistance and side effects of patients, which provides a new and effective treatment for cancer. Nagaraju et al. (2021) In addition, through the surface modification of nanoparticle structures and the integration of tumor diagnosis and treatment, nanomaterials can also be used for early diagnosis, imaging, precision therapy and smart anti-cancer drugs. Auría-Soro et al. (2019).

As an important endogenous trace element, iron is widely distributed in various tissues of human body to maintain biological health. In recent years, iron-based nanomaterials have been widely used in various fields of biomedicine. Xie et al. (2018) Through the precise control and surface modification of magnetic materials, nano-materials such as iron oxide nanoparticles have been widely used in the biomedical research field, as a contrast agent for drug delivery such as magnetic resonance magnetic resonance imaging hyperthermia drug delivery. Shen et al. (2017) Recently, with the development of nanomedicine, more and more IONPs have been used in the diagnosis and treatment of leukemia.

### 2.1 Preparation and modification of iron oxide nanoparticles

There are many methods for preparing magnetic iron oxide nanoparticles, such as physical methods, chemical methods and microbial technology. Wei and Wang (2013) The physical chemistry properties of the prepared magnetic ferroferric oxide nanoparticles (Fe_3_O_4_NPs) are closely related to the preparation methods. Laurent et al. (2008) Therefore, it is very important to choose a suitable method to synthesize magnetic nanoparticles; Song et al. (2019) Among the many methods, the most commonly used are chemical methods, which have lower synthesis costs and higher yields; Hu et al. (2018) Chemical methods of preparation of Fe_3_O_4_NPs include thermal decomposition method, hydrothermal method, co-precipitation method, micro-emulsion method; Sodipo and Aziz, (2016) The most common method for Fe_3_O_4_NPs preparation is co-precipitation. There are the detailed characteristics of these methods (Table 1).

**TABLE 1:** Comparison of the characteristics of different synthesis methods of IONPs.

Method name	Principle	Advantages	Disadvantages	Impact factors	Reference
Thermal decomposition method	The organic metal precursor is decomposed into metal oxide at high temperature or metallic elements	monodisperse and uniform in diameter	The product surface is hydrophobic and needs to be modified	Temperature, heating rate, reaction time	Lu et al. (2007)
Hydrothermal method	The hot solvent is converted into nanoparticles under high temperature and high pressure	simple operation, high crystallinity, good hydrophilicity and no need of surface modification	Reaction equipment is expensive, to the temperature, the pressure, the device request is high	Temperature, reaction time, type of solvent	Li et al., (2013)
Coprecipitation method	Ferric and ferric precipitates in alkaline solutions	Good hydrophilicity, simple procedure and high yield	Product size polydispersity	Temperature, pH, concentration, type of iron salt	Frimpong et al., (2010)
Microemulsion method	Surfactants and bases are added to form a microemulsion system in which the co-precipitation reaction takes place	size controlled, and the product has monodispersety	Toxic Surfactant	Emulsifier, surfactant concentration ratio, reaction temperature and time	Bai et al., (2007)

Although the preparation of Fe_3_O_4_NPs is mature, there are still several problems in production and application, such as the formation of aggregation due to the instability of nanoparticles. Mohammadi et al. (2013) Therefore, it is necessary to modify the surface of Fe_3_O_4_NPs. The appropriate surface modification has ability to improve the stability, targeting ability and biocompatibility of Fe_3_O_4_NPs nanoparticles (Arias et al., 2018). The various materials used to modify IONPs have different advantages and applications (Table 2).

**TABLE 2:** Characteristics of different modifying materials for IONPs.

Classification	Representative	Advantage	Application	Reference
Small molecules	Carboxylic acid, oleic acid	High hydrophilicity	Targeted Drug, vacuum sealing	Reguera et al., (2017)
Biomolecules	Nucleic acid, polypeptide, protein	High targeting	Targeted Drugs, immunotherapy, gene therapy	Banchelli et al., (2014)
Inorganic Materials	Au, AG, MOS2	High stability, simple preparation	Targeted drug delivery, multimodality imaging, and photothermal therapy	Li et al., (2015)
Mesoporous materials	Mesoporous silicon dioxide, organic metal frame MOFs	High hydrophilicity, high drug loading rate	Targeted drug delivery, fluorescence imaging	Fang et al., (2020)
Polymer	PEG, PVP, PEI	Long circulation, high Biocompatibility	Targeted drug delivery, bioimaging	Dehaini et al., (2016)

### 2.2 Iron-based nanoparticles and magnetic resonance imaging

Molecular imaging plays an important role in tumor detection and prognosis monitoring (Gordon et al., 2019). This technology has high accuracy and reliability in elucidating the process of tumor development and monitoring the condition of patients. Current imaging techniques include optical imaging (OI), X-ray computed tomography (CT), Positron emission tomography Single-photon emission computed tomography (PET SPECT) (Fonti et al., 2019), magnetic resonance imaging (MRI) and ultrasound (US) (Mammatas et al., 2015). Compared with other imaging methods, MRI has many advantages, such as non-invasive, high resolution, high soft tissue resolution and fast living imaging. It has become an important tool in clinical imaging diagnosis and disease monitoring (Chandarana et al., 2018).

The contrast agent can shorten the longitudinal and transverse relaxation time of the surrounding hydrogen protons, thus enhancing the signal contrast of the focus and improving the detection sensitivity (Shuvaev et al., 2021). MRI contrast media are usually divided into T_1_W_1_ and T_2_W_1_ (Kanda et al., 2015). Currently, most gadolinium-based T_1_ contrast agents are used clinically. However, gadolinium-based T_1_ contrast agents tend to dissociate *in vivo*, and free gadolinium ions can deposit in the kidneys and central nervous system, causing nephrogenic systemic fibrosis and neurotoxicity (McDonald et al., 2015). Therefore, for patients with renal dysfunction, gadolinium-based contrast agents may aggravate renal toxicity and even cause renal failure. Because of it is reported that iron-based nanoparticles have better biocompatibility than gadolinium-based contrast agents due to its unique magnetism and tiny size (Yang et al., 2012). Neuwelt E A et al. studied the effect of size on IONPs, and the results showed that when the size of ferrite nanoparticles is less than 5 nm, the decrease of magnetic moment can not only suppress the T_2_ effect, but also enhance the T_1_ signal effectively, which reflects the high T_1_ contrast enhancement performance (Neuwelt et al., 2009).

**FIGURE 2:**
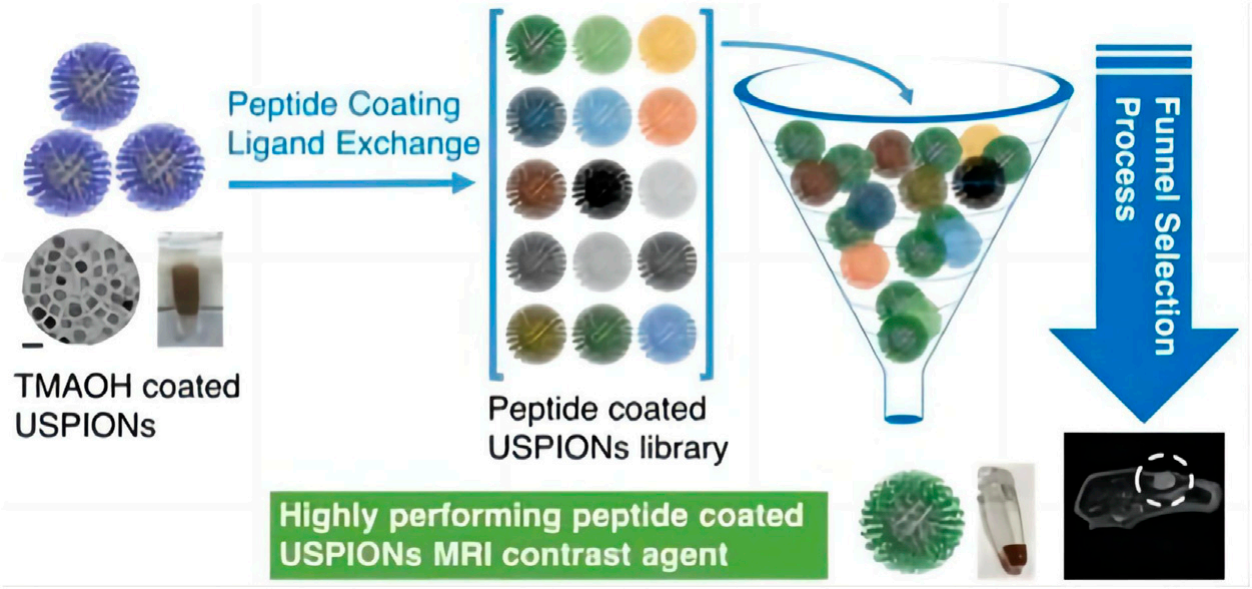
Synthesis process and application of 2PG-S*VVVT-PEG4-ol-coated USPIONs (Chee et al., 2018).

IONPs can be modified with biological material to improve the biocompatibility of the contrast medium. Chee, H et al. designed a short peptide and ligand library for enhancing MRI Biocompatibility, which was used to prepare 86 different peptide-coated ultra miniature superparamagnetic iron oxide nanoparticles (USPION) (Chee et al., 2018). Diphosphopeptide 2 PG-S *VVVT-PEG 4-OL was screened and found to provide the highest biocompatibility and performance for USPION, with no detectable toxicity or adhesion to living cells, and was comparable to commercially available contrast media, the peptide-coated USPION can be used to target the tumor, improve the contrast of the target site, and show better MRI performance (Figure 2). Compared with conventional MRI contrast agents, this kind of contrast agent with iron oxide nanoparticles has a higher overall safety and targeting function.

At present, MRI is mainly used to evaluate the prognosis of leukemia and to screen the complications caused by the treatment of leukemia (Mayerhoefer et al., 2020). Intracranial infection is a common central nervous system complication of leukemia. Leukemia patients, especially children, can be infected with microorganisms in the central nervous system due to the damage of immune system, damage of immune system caused by chemotherapy and persistent neutrophils (Lim et al., 2021). Previous intracranial infections were mainly diagnosed by clinical manifestations and laboratory examinations such as cerebrospinal fluid (CSF). Because of the similar clinical characteristics of these central nervous system complications, it is sometimes difficult to make a differential diagnosis by clinical laboratory tests, and the location and extent of the lesions can not be determined by CSF smears (Abdalkader et al., 2019). MRI has some advantages over laboratory tests in leukemia with central nervous system infection. Moreover, MRI can identify the location and extent of the lesion, on the other hand, the infection caused by different pathogenic microorganisms has different imaging features, therefore, MRI can help to diagnose infectious brain lesions caused by leukemia and differentiate other central system diseases (Mayerhoefer et al., 2020). Because of the ultra-small size and specific magnetic properties of iron oxide nanoparticles, iron nanoparticles have great potential as MRI contrast agents in the diagnosis of leukemia-related central system complications.

### 2.3 Magnetic hyperthermia

Magnetic hyperthermia was proposed by Gilchrist in 1957. On the basis of the research on the destruction of dog’s lymphoma tissues by magnetic materials, it was proved that magnetic materials could move and gather in the tumor tissues in a targeted manner, and generate heat to destroy the tumor tissue under the action of alternating magnetic field (Gilchrist et al., 1957). With the rise of nanotechnology, magnetic hyperthermia is developing constantly. Magnetic hyperthermia is based on the principle that tumor cells are different from normal cells in their tolerance to temperature, under the action of alternating magnetic field, magnetic nanoparticles produce magnetic hysteresis and relaxation, which leads to heat generation. Finally, the temperature in the tissue region is increased, and the tumor cells are destroyed (Carrey et al., 2011). Under the action of alternating magnetic field, magnetic nanoparticles produce hysteresis and relaxation, thus generating heat and increasing the temperature of tissue area (Hergt et al., 2009). The raised temperature range of 43°C–46°C induces injury of tumor cells, which undergo physiological changes leading to their apoptosis/necrosis, while normal cells are not affected (Spirou et al., 2018). At present, there are many researches on Magnetic hyperthermia therapy (MHT) in the field of solid tumor treatment, while there are few studies on MHT in the field of non-solid tumor treatment such as leukemia. Magnetic nanomaterials can be modified to functionalize the surface to target the tumor site (Carter et al., 2021). Several studies suggested that epithelial cell adhesion molecule (Ep-CAM) might be a marker reflecting the epithelial state of primary and systemic tumor cells, and the specific expression of Ep-CAM in leukemia cells could be used as an indicator to measure the metastasis of leukemia tumor cells (Zheng et al., 2017). Kim et al. fixed the antibody of epithelial cell adhesion molecule on the surface of magnetic nanoparticles (MNPs) to realize the specificity to leukemia cells. With EpCAM-MNPs hyperthermia in THP 1 cells and AKR mouse models, leukemic cell numbers were reduced by approximately 40% compared with control samples (Al Faruque et al., 2020). This experiment provides favorable supporting evidence for the treatment of leukemia with magnetic thermotherapy, so magnetic nanoparticles-mediated magnetic thermotherapy has a certain prospect in the treatment of leukemia.

## 3 Application of iron nanoparticles in leukemia

### 3.1 Iron-oxide nanoparticles and leukemia diagnosis

Unlike other diseases, based on the malignant proliferation and metastasis of cancer cells, one of the most effective treatments for cancer is to diagnose leukemia as early as possible. Traditional methods rely on the patient’s clinical symptoms, cytomorphology and cytogenetics to diagnose leukemia. However, traditional methods are less sensitive and leukemia cells can not be detected in the early stages of the disease. Therefore, it is necessary to develop a highly selective and sensitive method for the diagnosis of leukemia. Based on the advantages of good selectivity, high sensitivity, simple equipment and low price, biosensors have been used as diagnostic tools in drug detection, biomedical evaluation, environmental monitoring (Sharifi et al., 2019).

**FIGURE 3:**
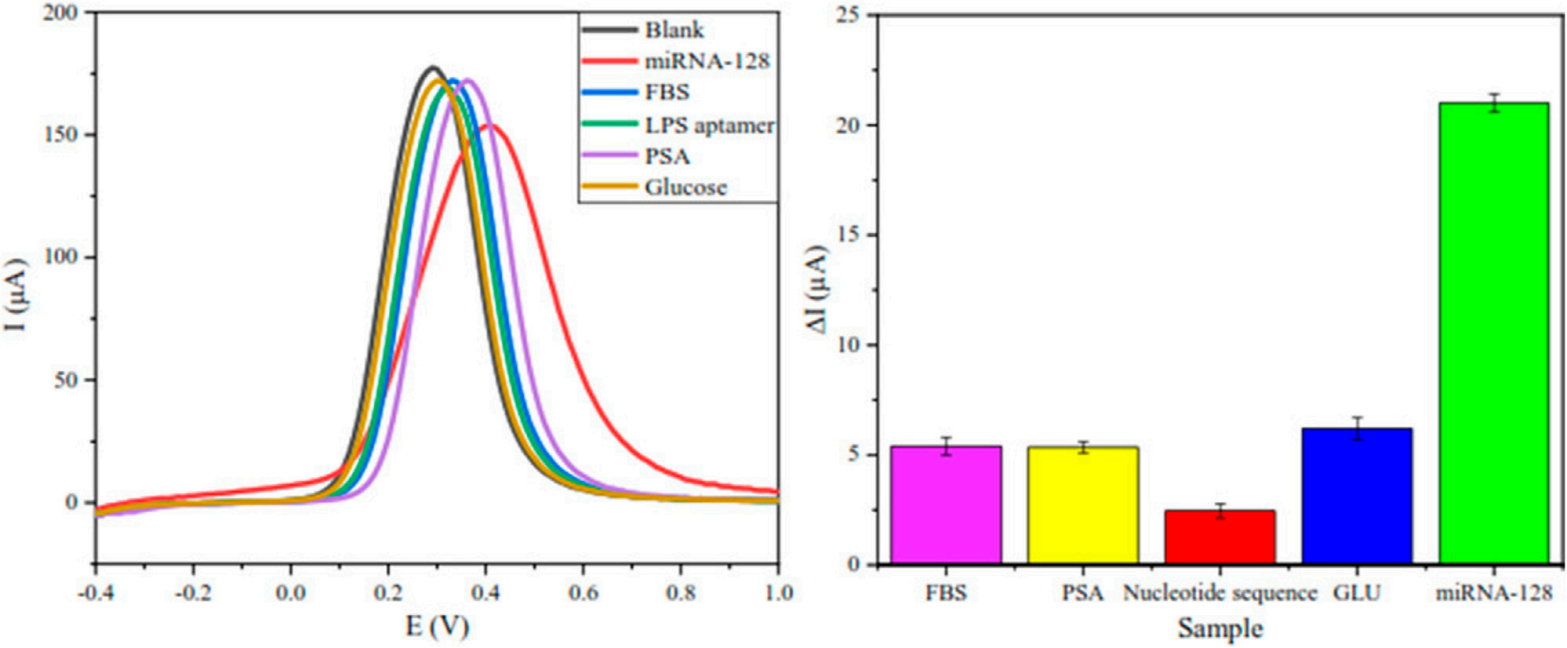
To confirm the high selectivity of the biosensor, besides miRNA-128, this biosensor was also documented for four other biomolecules that are in concentration in healthy humans, including LPS aptamer, glucose, PSA and BSA (Dinani et al., 2022).

Many studies have shown that the application of nanomaterials in traditional biosensors can improve the detection sensitivity, speed and selectivity. The combination of metal oxide nanoparticles and biosensor can not only increase the sensitivity, but also improve the signal-to-noise ratio (Kaushik et al., 2008). Dinani et al. (2022) constructed the first gold nanoparticle/magnetite/reduced graphene oxide (AuNPs/Fe_3_O_4_/RGO) adapter sensor for detecting the concentration of the leukemia biomarker miRNA-128. The electrical conductivity and the sensitivity of the sensor have been improved by this nanocomposite. Therefore, the quantitative determination of miRNA-128 by the biosensor is realized for the first time, which can be applied to the diagnosis and prognosis of acute lymphocyte leukemia (ALL). Because of the specific expression of miRNA-128 in ALL, the sensor can also be used for differential diagnosis of different types of leukemia, especially with AML, in which miRNA-128 is not expressed. (Figure 3).

Fe_3_O_4_ nanomaterials as metal oxide nanomaterials can increase the active surface area of the electrode surface and the electron transfer rate of redox, which leads to higher catalytic activity. Sgc8c linker can specifically bind to PTK7 which overexpresses in leukemia cells (Liu et al., 2019). Khoshfetrat and Mehrgardi (2017) first fixed the thiogenated sgc8c junction on the magnetic Fe_3_O_4_ nanoparticles (Apt-GMNPs) coated with gold nanoparticles, and then used N-doped graphene nanoplates as the detection electrode, an electrochemical joint sensor was successfully constructed to detect the selectivity and sensitivity of leukemic cells. It is reported that nitrogen-doped graphene had larger functional surface area, more active sites catalytic reactions and higher conductivity compared with graphene nanosheet (Wen et al., 2014). Moreover, gold coated MNPs as a separation tool have ability to decrease the signal-to-noise ratio in complex media. The unique magnetism of MNPs can be used not only in biosensors, but also in combination with gene detection technology to improve the sensitivity of leukemia detection (Manthawornsiri et al., 2016).

Recently, Magnetic separation technology has been discovered and applied to separate substances under magnetic field. Compared with other separation methods, such as centrifugation, electrophoresis and ultrafiltration, the advantages of magnetic separation involved of high specificity, mild reaction conditions, simple operation and low cost (Liu et al., 2020). In 1973, Fe_3_O_4_ NPs were firstly applied to the field of biological magnetic separation by Robinson et al. (1973). Nowadays, Fe_3_O_4_ NPs have been widely used in the separation of biological macromolecules such as cells, pathogens, nucleic acids and proteins (You et al., 2019). Rashid et al. (2020) prepared Fe_3_O_4_ NPs by coprecipitation, and coated the nanoparticles with SiO_2_. Finally, Fe_3_O_4_@SiO_2_ was functionalized to obtain Fe_3_O_4_@SiO_2_@PMIDA MNP. The nanocomposite is not only simple and inexpensive to fabricate, but also selectively isolates CD4^+^ T lymphocytes from human peripheral blood. Quynh et al. (2018) developed a novel Fe_3_O_4_/AG nanocomposite with core-shell structure and coupled anti-CD34 antibody to magnetic nanoparticles, which can collect CD34 + stem cells from bone marrow samples with high selectivity. The above researches on the application of magnetic separation in the separation of cells, which indicated that it is promising for the diagnosis of leukemia *via* magnetic separation technology.

### 3.2 Iron-oxide nanoparticles and leukemia treatment

#### 3.2.1 Improve the bioavailability of drugs

It is suggested that oral administration might be often regarded as common administration routes for the treatment of hematologic tumors (Liu, 2021). However, due to low water solubility and low oral absorption of most therapeutic drugs, it is necessary to increase the oral dose of cytotoxic drugs in order to enhance the blood concentration of drugs, which also causing damage of normal cells to a certain extent (Kantarjian et al., 2021). Therefore, it is important to discover a method to improve the bioavailability of drugs.

Homoharringtonine (HHT) can induce apoptosis by activating Caspase-3 and decreasing the expression of BCL-2, which can be used in tyrosine-kinase inhibitor myeloid leukemia (Chen et al., 2019). However, poor water solubility and serious adverse reactions limit its clinical application. In order to improve the bioavailability of HHT and reduce the adverse reactions, HHT-MNP-Fe_3_O_4_ nanoparticles were prepared as drug delivery nanoparticles (Li et al., 2020). The results showed that HHT delivered by MNP-Fe_3_O_4_ could not only inhibit the growth of myeloid leukemia cell, but also inhibit the proliferation of leukemia cells *in vivo* and *in vitro*, and induce apoptosis of leukemia cells on a wider range. The reason why HHT-MNP-Fe_3_O_4_ can improve the curative effect may be that the magnetic nanoparticles downregulate the expression of myeloid leukemia-1, inhibit the activation of cal-pain I and poly-ADP- ribose polymerase, and thus induce apoptosis of leukemia cells. Like HHT, genistein has anti-tumor potential. However, due to its poor water solubility, the effective doses of genistein will have more side effects when used to treat blood tumors. Chouhan et al. (2021); Ghasemi Goorbandi et al. (2020) prepared Fe_3_O_4_-CMC-genistein by covalently modifying Fe_3_O_4_ nanoparticles with Genistein. This study showed that the release rate of the synthesized Fe_3_O_4_-CMC-genistein was very slow, and compared with genistein, the synthesized Fe_3_O_4_-CMC- genistein showed a higher inhibition rate, especially at 72 h.

Most chemotherapeutic drugs used to treat leukemia have a short half-life and therefore do not reach effective therapeutic concentrations in the bloodstream for long enough to produce a lasting therapeutic effect. For example, the half-life of a chlorambucil is only 1–2 h, and the half-life of a mercaptopurine is 90 min (Sang et al., 2022). Fe_3_O_4_ nanoparticles can overcome this disadvantage. Chemotherapeutic drugs are encapsulated in nanoparticles to slow or control drug release and thereby improve drug bioavailability. Chloramb is used as an alkylating agent in the chemotherapy of chronic lymph leukemia (CLL) and chronic myeloid leukemia (CML). CS-IONPs with core-shell structure were prepared by Hussein-Al-Ali et al. (2021) Chloramb-CS-IONPs were synthesized by ionic gelation method using CS-IONPs as carriers of chlorambucil with 19% loading rate of chloramb. In this study, Chloramb-loaded IONPs reduced cancer cell viability in a leukemia cell line (WEHI) better than Chloramb. Furthermore, the release of Chloramb from the drug-loaded complex has proved to be a controlled release behavior. Chloramb-CS-IONPs provided a new way to improve the bioavailability of chloramphenylene, thus providing a new idea for the treatment of leukemia with Chloramb. Dorniani et al. (2014) used a co-precipitation method to mercaptopurine iron oxide nanoparticles coated with PEG. By simulating the pH of the stomach and blood, the drug-release behavior of the nanoparticles is controlled, thus the anti-leukemia effect of WEHI-3B can be maintained.

#### 3.2.2 Improve the efficiency of treatment

The clinical use of systemic chemotherapy for leukemia is often hampered by cancer cells and the multiple drug resistance of anticancer drugs. The process of multidrug resistance (MDR) development is very complex, which is related to transporter overexpression, enhanced xenobiotics metabolism, DNA repair ability changes, genetic factors and so on Bukowski et al. (2020). Nano-agents will play an important role in the treatment of multi-drug resistant tumors because they can not only carry multiple chemotherapeutic agents and bioactive components, but also overcome multiple mechanisms of MDR.

To reverse MDR and minimize serious adverse effects of systemic chemotherapy, Wang et al. (2011) used oleic acid-coated IONPs as a co-delivery vehicle for DNR and Br Tet, named DNR/Br Tet-MNPs. The effect of the drug-loaded nanoparticles on the apoptosis of drug-resistant human leukemia K562/A02 cells was studied. The study showed that DNR/Br Tet-MNPs could deliver DNR to drug-resistant cells better than DNR and Br Tet Solution. The reason may be that the drug-loaded nanoparticles decrease the expression of BCL-2 and increase the expression of Caspase 3, thus increasing the apoptosis of drug-resistant leukemia cells.

**FIGURE 4:**
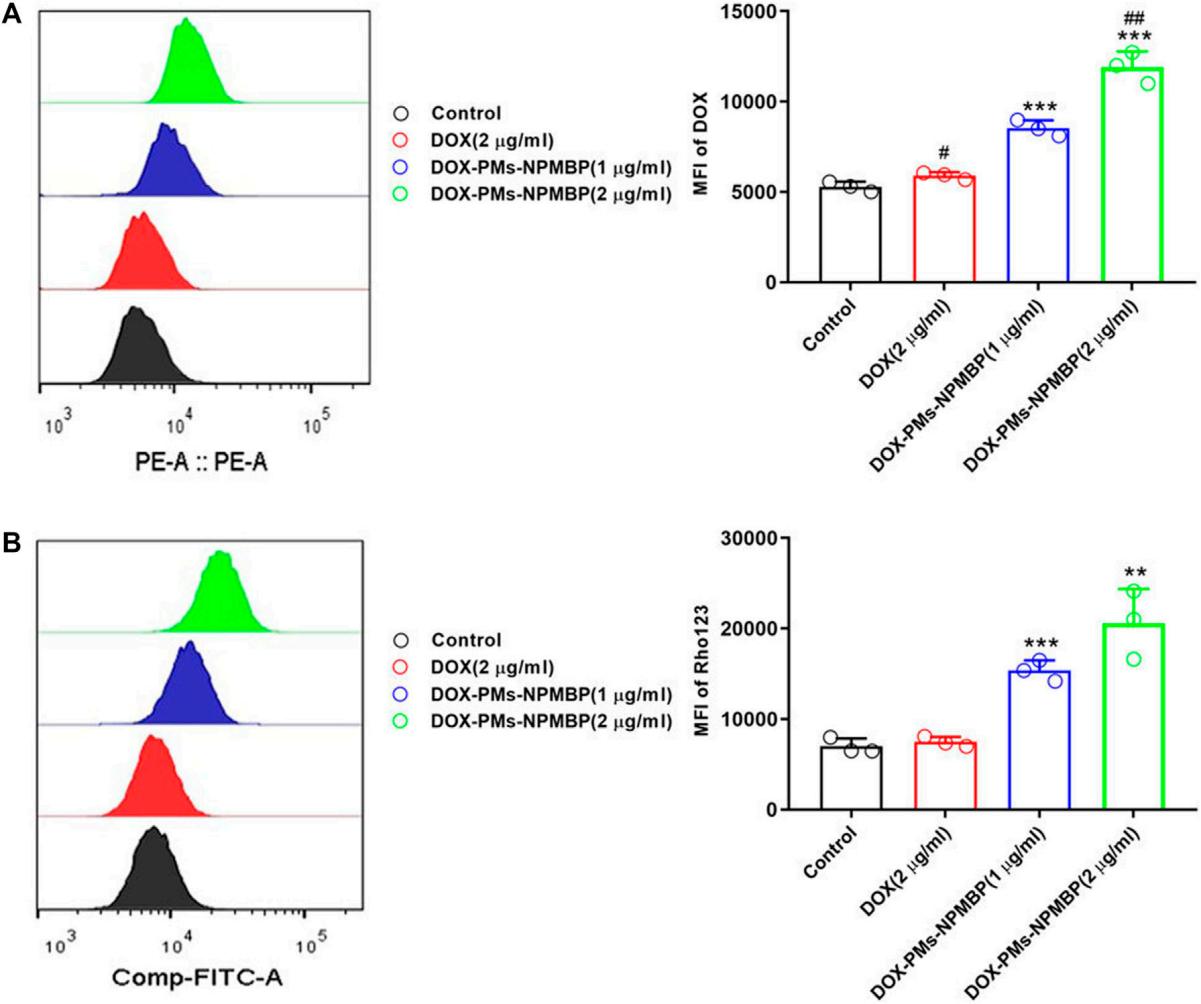
DOX-PMs-NPMBP promotes the intracellular retention of DOX in DOX-resistant cells. **(A)** The intracellular retention of DOX was assessed by flow cytometry in Nalm6/DOX cells after different treatments. **(B)** The MFI of Rhodamine123in Nalm123/ADR cells was assessed by flow cytometry analysis through FITC channels (Gan et al., 2021).

Doxorubicin (DOX) is a commonly used drug to treat acute leukemia, but its application is limited because of drug resistance. Studies have shown that over-expression of NPM can lead to multidrug resistance of ALL, and the engineered recombinant NPM binding protein (NPMBP) can knock out NPM by RNA interference, thus reversing multidrug resistance of leukemia cells (Wang et al., 2015). Gan D and his colleagues (Gan et al., 2021) assembled DOX and NPMBP into polymer nanomicelles called DOX-PMs-NPMBP. Compared with DOX, DOX-PMs-NPMBP could increase the drug retention capacity of DOX-resistant cells and induce G0/G1 phase arrest of drug-resistant cells, thus achieving a more significant anti-leukemia effect. In addition, the study showed that the multidrug resistance mechanism of DOX-PMs-NPMBP nanoparticles system was proved by the Rho123 outflow test by regulating the functional activity of P-glycoprotein (P-gp). Overexpression of P-gp in drug-resistant cells increases drug efflux, resulting in a decrease in intracellular drug concentration. The new DOX-PMs-NPMBP nanoparticle system can significantly regulate the functional activity of P-gp, thus reducing the efflux of Rho123 mediated by drug pump P-gp in drug-resistant ALL cells (Figure 4). These results suggest that the classical MDR phenotype-dependent mechanism is involved in the effect of DOX-PMs-NPMBP on DOX-resistant cells.

**FIGURE 5:**
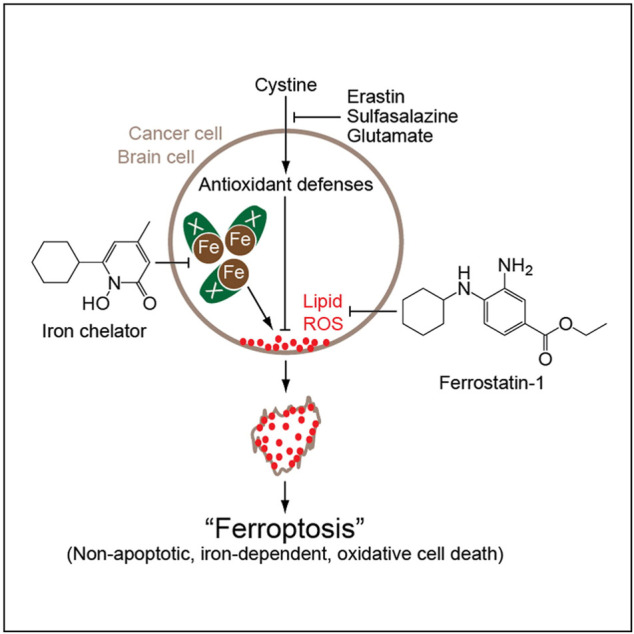
Mechanism of action of iron death (Dixon et al., 2012).

IONPs can trigger the Fenton reaction, a chemical reaction in which divalent and/or ferric can convert hydrogen peroxide into hydroxyl radicals. The Fenton reaction produces reactive oxygen species (ROS) and mediates lipid peroxidation, which can lead to ferroptosis. Shen et al. (2018) Ferroptosis, discovered and named by Dixon et al. (2012), is a new form of iron-dependent cell death that differs from apoptosis, cell necrosis, and autophagy (Figure 5). The biochemical mechanisms of ferroptosis include the production of lethal ROS, lipid peroxidation and intracellular iron accumulation, which in turn produce large amounts of oxygen free radicals. When the steady-state imbalance of ROS production and degradation occurs, the cell’s own antioxidant capacity decreases to a point where it is not sufficient to clear the excessive accumulation of lipid ROS. Hydroxyl radicals and ROS can attack structures such as DNA, proteins and cell membranes, and thus destroy the structure and function of cells, causing ferroptotic cell death. Chen et al. (2021).

Trujillo-Alonso et al. (2019) found that iron oxide nanoparticles approved by the FDA for the treatment of iron deficiency can be used to treat leukemia with low ferroportin (FPN) expression. Iron oxide nanoparticles can aggravate the reduction of cellular iron efflux and increase intracellular iron content, thus aggravating the increase of intracellular ROS level. Because FPN is highly expressed in normal hematopoietic cells, and iron oxide nanoparticles do not cause severe toxic and side effects in normal hematopoietic cells. Therefore, iron oxide nanoparticles are relatively safe in the treatment of this kind of leukemia.

Dou et al. (2021) found that the combination of Fe_3_O_4_ nanoparticles with cytarabine (Ara-C) inhibited leukemia stem cell (LSC) more than Ara-C alone. They analyzed changes in ROS-related substances such as gp91-phox and SOD1 by measuring ROS levels in LSC and non-LSC under different conditions. It was demonstrated that IONPs and Ara-C might induce LSC apoptosis due to increased ROS levels. Therefore, iron oxide nanoparticles can regulate the oxidative damage and defense balance of cells through the mechanism of iron death, which has a great prospect in the treatment of leukemia.

## 4 Conclusion and prospects

With the development of medical science and technology, the traditional way of drug delivery will be innovated. Due to the convenience and biocompatibility of IONPs, it has a broad application prospects in clinical transformation. The rapid development of nanotechnology has laid a solid foundation for the diagnosis and treatment of leukemia, and more and more application of IONPs in the treatment of leukemia. Furthermore, IONPs are widely used in tumor imaging, magnetic hyperthermia and biomacromolecule magnetic separation because of their magnetic properties. More importantly, IONPs can not only achieve synergistic drug delivery, but also combine with other therapeutic methods to achieve better therapeutic effects, which has become one of the hot research directions.

However, the application of iron oxide nanoparticles in the diagnosis and treatment of leukemia still needs to overcome huge obstacles in the clinical transformation process. 1) At present, there is still a lack of high-yield and high-safety methods for the preparation of iron oxide nanoparticles. Although some modified materials have been studied, the synthetic standard of modified materials and whether other new materials can be used for modification are still an urgent problem to be solved. 2) Lack of detailed toxicity test results of iron oxide nanoparticles. Although iron oxide nanoparticles are biocompatible compared to other materials, the long-term side effects of iron oxide nanoparticles on normal cells, tissues and organs need to be further determined, and carefully study the metabolism of this material and degradation of the impact on the body. 3) The detailed mechanism of iron oxide nanoparticles involved in regulating the metabolism of leukemic cells is not completely clear. 4) For the application of IONPs in the treatment of leukemia, most of current studies are devoted to the examination of the drug-carrying function of IONPs, and there are fewer applications regarding the magnetic properties of IONPs, such as magnetic hyperthermia and magnetic separation. 6) The clinical transformation of iron oxide nanoparticles in leukemia has been very slow, and only a small number of studies have been carried out in clinical trials. 7) The design of iron oxide nanoparticles with imaging, diagnosis and therapy, and the realization of multi-treatment of leukemia, may be one of the hot research directions in the future.

These questions may inspire future research into the development and improvement of IONPs, providing better opportunities for the treatment of leukemia. In summary, combining multidisciplinary knowledge and research tools to explore effective therapies for leukemia and develop highly biocompatibility, highly targeted iron oxide nanoparticles will be the focus. It is believed that with the joint efforts of researchers, IONPs may be further applied in the clinical treatment of leukemia.

## References

[B1] Abdalkader M. Xie J. Cervantes-Arslanian A. Takahashi C. Mian A. Z. (2019). Imaging of intracranial infections. Seminars Neurology 39, 322–333. Thieme Medical Publishers. 10.1055/s-0039-1693161 31378868

[B2] Al Faruque H. Choi E.-S. Lee H.-R. Kim J.-H. Park S. Kim E. (2020). Targeted removal of leukemia cells from the circulating system by whole-body magnetic hyperthermia in mice. Nanoscale 12, 2773–2786. 10.1039/c9nr06730b 31957767

[B3] Alphandéry E. (2019). Biodistribution and targeting properties of iron oxide nanoparticles for treatments of cancer and iron anemia disease. Nanotoxicology 13, 573–596. 10.1080/17435390.2019.1572809 30938215

[B4] Alsalem M. A. Zaidan A. A. Zaidan B. B. Hashim M. Madhloom H. T. Azeez N. D. (2018). A review of the automated detection and classification of acute leukaemia: Coherent taxonomy, datasets, validation and performance measurements, motivation, open challenges and recommendations. Comput. methods programs Biomed. 158, 93–112. 10.1016/j.cmpb.2018.02.005 29544792

[B5] Arias L. S. Essan J. P. Vieira A. P. M. Lima T. M. T. Delbem A. C. B. Monteiro D. R. (2018). Iron oxide nanoparticles for biomedical applications: A perspective on synthesis, drugs, antimicrobial activity, and toxicity. Antibiotics 7, 46. 10.3390/antibiotics7020046 29890753 PMC6023022

[B6] Auría-Soro C. Nesma T. Juanes-Velasco P. Landeira-Viñuela A. Fidalgo-Gomez H. Acebes-Fernandez V. (2019). Interactions of nanoparticles and biosystems: Microenvironment of nanoparticles and biomolecules in nanomedicine. Nanomaterials 9, 1365. 10.3390/nano9101365 31554176 PMC6835394

[B7] Bai F. Wang D. Huo Z. Chen W. Liu L. Liang X. (2007). A versatile bottom‐up assembly approach to colloidal spheres from nanocrystals. Angew. Chem. 119, 6650–6653. 10.1002/anie.200701355 17661295

[B8] Banchelli M. Nappini S. Montis C. Bonini M. Canton P. Berti D. (2014). Magnetic nanoparticle clusters as actuators of ssDNA release. Phys. Chem. Chem. Phys. 16, 10023–10031. 10.1039/c3cp55470h 24487734

[B9] Bodet-Milin C. Kraeber-Bodéré F. Eugène T. Guérard F. Gaschet J. Bailly C. (2016). Radioimmunotherapy for treatment of acute leukemia. Seminars Nucl. Med. 46, 135–146. Elsevier. 10.1053/j.semnuclmed.2015.10.007 26897718

[B10] Bukowski K. Kciuk M. Kontek R. (2020). Mechanisms of multidrug resistance in cancer chemotherapy. Int. J. Mol. Sci. 21, 3233. 10.3390/ijms21093233 32370233 PMC7247559

[B11] Carrey J. Mehdaoui B. Respaud M. (2011). Simple models for dynamic hysteresis loop calculations of magnetic single-domain nanoparticles: Application to magnetic hyperthermia optimization. J. Appl. Phys. 109, 083921. 10.1063/1.3551582

[B12] Carter T. J. Agliardi G. Lin F.‐Y. Ellis M. Jones C. Robson M. (2021). Potential of magnetic hyperthermia to stimulate localized immune activation. Small 17, 2005241. 10.1002/smll.202005241 33734595

[B13] Chandarana H. Wang H. Tijssen R. H. N. IndraDas J. (2018). Emerging role of MRI in radiation therapy. J. Magnetic Reson. Imaging 48, 1468–1478. 10.1002/jmri.26271 PMC6986460 30194794

[B14] Chee H. L. Gan C. R. R. Ng M. Low L. Fernig D. G. KishoreBhakoo K. (2018). Biocompatible peptide-coated ultrasmall superparamagnetic iron oxide nanoparticles for *in vivo* contrast-enhanced magnetic resonance imaging. ACS Nano 12, 6480–6491. 10.1021/acsnano.7b07572 29979569

[B15] Chen S.‐J. Zhou G.‐B. (2012). Targeted therapy: The new lease on life for acute promyelocytic leukemia, and beyond. IUBMB life 64, 671–675. 10.1002/iub.1055 22714999

[B16] Chen X. Kang R. Guido K. Tang D. (2021). Broadening horizons: The role of ferroptosis in cancer. Nat. Rev. Clin. Oncol. 18, 280–296. 10.1038/s41571-020-00462-0 33514910

[B17] Chen X.-J. Zhang W.-N. Chen B. Xi W.-D. Lu Y. Huang J.-Y. (2019). Homoharringtonine deregulates MYC transcriptional expression by directly binding NF-κB repressing factor. Proc. Natl. Acad. Sci. 116, 2220–2225. 10.1073/pnas.1818539116 30659143 PMC6369765

[B18] Chouhan R. S. Horvat M. Ahmed J. Alhokbany N. Alshehri S. M. Gandhi S. (2021). Magnetic nanoparticles—a multifunctional potential agent for diagnosis and therapy. Cancers 13, 2213. 10.3390/cancers13092213 34062991 PMC8124749

[B19] Curtis R. E. Boice J. D. Jr Stovall M. Bernstein L. Greenberg R. S. Flannery J. T. (1992). Risk of leukemia after chemotherapy and radiation treatment for breast cancer. N. Engl. J. Med. 326, 1745–1751. 10.1056/NEJM199206253262605 1594016

[B20] Dehaini D. Fang R. H. Luk B. T. Pang Z. Hu C. M. Kroll A. V. (2016). Ultra-small lipid–polymer hybrid nanoparticles for tumor-penetrating drug delivery. Nanoscale 8, 14411–14419. 10.1039/c6nr04091h 27411852 PMC4977227

[B21] Dinani H. S. Pourmadadi M. Yazdian F. Rashedi H. Ebrahimi S. A. S. Shayeh J. S. (2022). Fabrication of Au/Fe3O4/RGO based aptasensor for measurement of miRNA‐128, a biomarker for acute lymphoblastic leukemia (ALL). Eng. Life Sci. 22, 519–534. 10.1002/elsc.202100170 35936072 PMC9349134

[B22] Dixon S. J. Lemberg K. M. Lamprecht M. R. Skouta R. Zaitsev E. M. Gleason C. E. (2012). Ferroptosis: An iron-dependent form of nonapoptotic cell death. Cell 149, 1060–1072. 10.1016/j.cell.2012.03.042 22632970 PMC3367386

[B23] Dorniani D. Kura A. U. Hussein-Al-Ali S. H. bin Hussein M. Z. Fakurazi S. Shaari A. H. (2014). Release behavior and toxicity profiles towards leukemia (WEHI-3B) cell lines of 6-mercaptopurine-PEG-coated magnetite nanoparticles delivery system. Sci. World J. 2014, 972501. 10.1155/2014/972501 PMC4034514 24895684

[B24] Dou J. Li L. Guo M. Mei F. Zheng D. Xu H. (2021). Iron oxide nanoparticles combined with cytosine arabinoside show anti-leukemia stem cell effects on acute myeloid leukemia by regulating reactive oxygen species. Int. J. nanomedicine 16, 1231–1244. 10.2147/IJN.S278885 33633448 PMC7900778

[B25] El-Sherbiny I.M. Elbaz N. M. Sedki M. Elgammal A. Yacoub M. H. (2017). Magnetic nanoparticles-based drug and gene delivery systems for the treatment of pulmonary diseases. Nanomedicine 12, 387–402. 10.2217/nnm-2016-0341 28078950

[B26] Fang C. Deng Z. Cao G. Chu Q. Wu Y. Xiang L. (2020). Co–ferrocene MOF/glucose oxidase as cascade nanozyme for effective tumor therapy. Adv. Funct. Mater. 30, 1910085. 10.1002/adfm.201910085

[B27] Fonti R. Conson M. Del Vecchio S. (2019). PET/CT in radiation oncology. Seminars Oncol. 46, 202–209. Elsevier. 10.1053/j.seminoncol.2019.07.001 31378377

[B28] Frimpong R. A. Dou J. Pechan M. Hilt J. Z. (2010). Enhancing remote controlled heating characteristics in hydrophilic magnetite nanoparticles via facile co-precipitation. J. Magnetism Magnetic Mater. 322, 326–331. 10.1016/j.jmmm.2009.09.050

[B29] Gan D. Chen Y. Wu Z. Luo L. Yirga S. K. Zhang N. (2021). Doxorubicin/Nucleophosmin binding protein-conjugated nanoparticle enhances anti-leukemia activity in acute lymphoblastic leukemia cells *in vitro* and *in vivo* . Front. Pharmacol. 12, 607755. 10.3389/fphar.2021.607755 34122059 PMC8193937

[B30] Gardin C. Pautas C. Fournier E. Itzykson R. Lemasle E. Bourhis J.-H. (2020). Added prognostic value of secondary AML-like gene mutations in ELN intermediate-risk older AML: ALFA-1200 study results. Blood Adv. 4, 1942–1949. 10.1182/bloodadvances.2019001349 32380535 PMC7218423

[B31] Ghasemi Goorbandi R. Mohammadi M. R. Malekzadeh K. (2020). Synthesizing efficacious genistein in conjugation with superparamagnetic Fe3O4 decorated with bio-compatible carboxymethylated chitosan against acute leukemia lymphoma. Biomaterials Res. 24, 9–13. 10.1186/s40824-020-00187-2 PMC7082912 32206338

[B32] Gilchrist R. K. Medal R. Shorey W. D. Hanselman R. C. Parrott J. C. Taylor C. B. (1957). Selective inductive heating of lymph nodes. Ann. Surg. 146, 596–606. 10.1097/00000658-195710000-00007 13470751 PMC1450524

[B33] Gordon O. Ruiz-Bedoya C. A. Ordonez A. A. Tucker E. W. Jain S. K. (2019). Molecular imaging: A novel tool to visualize pathogenesis of infections *in situ* . MBio 10, e00317–e00319. 10.1128/mBio.00317-19 31662452 PMC6819656

[B34] Hergt R. Dutz S. Zeisberger M. (2009). Validity limits of the Néel relaxation model of magnetic nanoparticles for hyperthermia. Nanotechnology 21, 015706. 10.1088/0957-4484/21/1/015706 19946160

[B35] Hu Y. Mignani S. Majoral J.-P. Shen M. Shi X. (2018). Construction of iron oxide nanoparticle-based hybrid platforms for tumor imaging and therapy. Chem. Soc. Rev. 47, 1874–1900. 10.1039/c7cs00657h 29376542

[B36] Hussein-Al-Ali S. H. Hussein M. Z. Bullo S. Arulselvan P. (2021). Chlorambucil-iron oxide nanoparticles as a drug delivery system for leukemia cancer cells. Int. J. nanomedicine 16, 6205–6216. 10.2147/IJN.S312752 34526768 PMC8435621

[B37] Kadia T. M. Ravandi F. Cortes J. Kantarjian H. (2015). Toward individualized therapy in acute myeloid leukemia: A contemporary review. JAMA Oncol. 1, 820–828. 10.1001/jamaoncol.2015.0617 26181162

[B38] Kanda T. Fukusato T. Matsuda M. Toyoda K. Oba H. Kotoku J. (2015). Gadolinium-based contrast agent accumulates in the brain even in subjects without severe renal dysfunction: Evaluation of autopsy brain specimens with inductively coupled plasma mass spectroscopy. Radiology 276, 228–232. 10.1148/radiol.2015142690 25942417

[B39] Kantarjian H. Kadia T. DiNardo C. Daver N. Borthakur G. Jabbour E. (2021). Acute myeloid leukemia: Current progress and future directions. Blood cancer J. 11, 41. 10.1038/s41408-021-00425-3 33619261 PMC7900255

[B40] Kantarjian H. Ravandi F. O'Brien S. Cortes J. Faderl S. Garcia-Manero G. (2010). Intensive chemotherapy does not benefit most older patients (age 70 years or older) with acute myeloid leukemia. Blood, J. Am. Soc. Hematol. 116, 4422–4429. 10.1182/blood-2010-03-276485 PMC4081299 20668231

[B41] Kaushik A. Khan R. Solanki P. R. Pandey P. Alam J. Ahmad S. (2008). Iron oxide nanoparticles–chitosan composite based glucose biosensor. Biosens. Bioelectron. 24, 676–683. 10.1016/j.bios.2008.06.032 18692384

[B42] Khoshfetrat S. M. Mehrgardi M. A. (2017). Amplified detection of leukemia cancer cells using an aptamer-conjugated gold-coated magnetic nanoparticles on a nitrogen-doped graphene modified electrode. Bioelectrochemistry 114, 24–32. 10.1016/j.bioelechem.2016.12.001 27992855

[B43] Laurent S. Forge D. Port M. Roch A. Robic C. Vander Elst L. (2008). Magnetic iron oxide nanoparticles: Synthesis, stabilization, vectorization, physicochemical characterizations, and biological applications. Chem. Rev. 108, 2064–2110. 10.1021/cr068445e 18543879

[B44] Lee D.-E. Koo H. Kwon I. C. Ryu J. H. Kim K. Kwon I. C. (2012). Multifunctional nanoparticles for multimodal imaging and theragnosis. Chem. Soc. Rev. 41, 2656–2672. 10.1039/c2cs15261d 22189429

[B45] Li C. Dong L. Su R. Bi Y. Qing Y. Deng X. (2020). Homoharringtonine exhibits potent anti-tumor effect and modulates DNA epigenome in acute myeloid leukemia by targeting SP1/TET1/5hmC. Haematologica 105, 148–160. 10.3324/haematol.2018.208835 30975912 PMC6939512

[B46] Li J. Hu Y. Jia Y. Wei P. Sun W. Shen M. (2015). Hyaluronic acid-modified Fe3O4@ Au core/shell nanostars for multimodal imaging and photothermal therapy of tumors. Biomaterials 38, 10–21. 10.1016/j.biomaterials.2014.10.065 25457979

[B47] Li J. Zheng L. Cai H. Sun W. Shen M. Zhang G. (2013). Polyethyleneimine-mediated synthesis of folic acid-targeted iron oxide nanoparticles for *in vivo* tumor MR imaging. Biomaterials 34, 8382–8392. 10.1016/j.biomaterials.2013.07.070 23932250

[B48] Li K. Nejadnik H. Daldrup-Link H. E. (2017). Next-generation superparamagnetic iron oxide nanoparticles for cancer theranostics. Drug Discov. today 22, 1421–1429. 10.1016/j.drudis.2017.04.008 28454771 PMC5610947

[B49] Lim E. A. Gnanadurai R. Ruffle J. K. Lee H. Miller R. F. Hyare H. (2021). Neuroimaging of CNS infection in haematological malignancy: Important signs and common diagnostic pitfalls. Clin. Radiol. 76, 470.e1–470.e12. 10.1016/j.crad.2021.01.009 33610289

[B50] Liu H. (2021). Emerging agents and regimens for AML. J. Hematol. Oncol. 14, 49. 10.1186/s13045-021-01062-w 33757574 PMC7989091

[B51] Liu M. Ma W. Li Q. Zhao D. Shao X. Huang Q. (2019). Aptamer‐targeted DNA nanostructures with doxorubicin to treat protein tyrosine kinase 7‐positive tumours. Cell Prolif. 52, e12511. 10.1111/cpr.12511 30311693 PMC6430458

[B52] Liu S. Yu B. Wang S. Shen Y. Cong H. (2020). Preparation, surface functionalization and application of Fe3O4 magnetic nanoparticles. Adv. colloid Interface Sci. 281, 102165. 10.1016/j.cis.2020.102165 32361408

[B53] Ljungman P. de la Camara R. Robin C. Crocchiolo R. Hermann E. Hill J. A. (2019). Guidelines for the management of cytomegalovirus infection in patients with haematological malignancies and after stem cell transplantation from the 2017 European Conference on Infections in Leukaemia (ECIL 7). Lancet Infect. Dis. 19, e260–e272. 10.1016/S1473-3099(19)30107-0 31153807

[B54] Lo S.-C. Pripuzova N. Li B. Komaroff A. L. Hung G.-C. Wang R. (2010). Detection of MLV-related virus gene sequences in blood of patients with chronic fatigue syndrome and healthy blood donors. Proc. Natl. Acad. Sci. 107, 15874–15879. 10.1073/pnas.1006901107 20798047 PMC2936598

[B55] Lu A.‐H. Salabas E. Schüth F. (2007). Magnetic nanoparticles: Synthesis, protection, functionalization, and application. Angew. Chem. Int. Ed. 46, 1222–1244. 10.1002/anie.200602866 17278160

[B56] Mammatas L. H. Verheul H. M. W. Harry Hendrikse N. Yaqub M. Lammertsma A. A. Menke-van der Houven van Oordt C. W. (2015). Molecular imaging of targeted therapies with positron emission tomography: The visualization of personalized cancer care. Cell. Oncol. 38, 49–64. 10.1007/s13402-014-0194-4 PMC13004269 25248503

[B57] Manthawornsiri Y. Polpanich D. Yamkamon V. Thiramanas R. Hongeng S. Rerkamnuaychoke B. (2016). Magnetic nanoparticles PCR enzyme‐linked gene assay for quantitative detection of BCR/ABL fusion gene in chronic myelogenous leukemia. J. Clin. Laboratory Analysis 30, 534–542. 10.1002/jcla.21899 PMC6807159 26667895

[B58] Mayerhoefer M. E. Archibald S. J. Messiou C. Staudenherz A. Berzaczy D. Schöder H. (2020). MRI and PET/MRI in hematologic malignancies. J. Magnetic Reson. Imaging 51, 1325–1335. 10.1002/jmri.26848 PMC7217155 31260155

[B59] McDonald R. J. McDonald J.r S. Kallmes D. F. Jentoft M. E. Murray D. L. KentThielen R. (2015). Intracranial gadolinium deposition after contrast-enhanced MR imaging. Radiology 275, 772–782. 10.1148/radiol.15150025 25742194

[B60] Mohammadi A. Barikani M. Barmar M. (2013). Effect of surface modification of Fe 3 O 4 nanoparticles on thermal and mechanical properties of magnetic polyurethane elastomer nanocomposites. J. Mater. Sci. 48, 7493–7502. 10.1007/s10853-013-7563-7

[B61] Na H. B. Song C. Hyeon T. (2009). Inorganic nanoparticles for MRI contrast agents. Adv. Mater. 21, 2133–2148. 10.1002/adma.200802366

[B62] Nagaraju G. P. Srivani G. Dariya B. Chalikonda G. Farran B. Kumar Behera S. (2021). Nanoparticles guided drug delivery and imaging in gastric cancer. Seminars Cancer Biol. 69, 69–76. Elsevier. 10.1016/j.semcancer.2020.01.006 31954835

[B63] Neuwelt E. A. Hamilton B. E. Varallyay C. G. Rooney W. R. Edelman R. D. Jacobs P. M. (2009). Ultrasmall superparamagnetic iron oxides (USPIOs): A future alternative magnetic resonance (MR) contrast agent for patients at risk for nephrogenic systemic fibrosis (NSF)? Kidney Int. 75, 465–474. 10.1038/ki.2008.496 18843256 PMC2643331

[B64] Newell L. F. Cook R. J. (2021). Advances in acute myeloid leukemia. Bmj 375, n2026. 10.1136/bmj.n2026 34615640

[B65] Pant M. Bhatt V. R. (2017). Early mortality and survival in older adults with acute myeloid leukemia. Future Med. 6 (3), 61–63. 10.2217/ijh-2017-0013 PMC6171990 30302225

[B66] Patra J. K. Das G. Fernandes Fraceto L. Campos E. V. R. Rodriguez-Torres M. P. Acosta-Torres L. S. (2018). Nano based drug delivery systems: Recent developments and future prospects. J. nanobiotechnology 16, 71–33. 10.1186/s12951-018-0392-8 30231877 PMC6145203

[B67] Pavlů J. Apperley J. F. (2013). Allogeneic stem cell transplantation for chronic myeloid leukemia. Curr. Hematol. malignancy Rep. 8, 43–51. 10.1007/s11899-012-0149-7 23275177

[B68] Peccatori J. Ciceri F. (2010). Allogeneic stem cell transplantation for acute myeloid leukemia. Haematologica 95, 857–859. 10.3324/haematol.2010.023184 20513804 PMC2878779

[B69] Quynh L. M. Dung C. T. Mai B. T. Huy H. V. Loc N. Q. Hoa N. Q. (2018). Development of Fe3O4/Ag core/shell-based multifunctional immunomagnetic nanoparticles for isolation and detection of CD34+ stem cells. J. Immunoass. Immunochem. 39, 308–322. 10.1080/15321819.2018.1488725 29995570

[B70] Rashid Z. Shokri F. Abbasi A. Khoobi M. Zarnani A.-H. (2020). Surface modification and bioconjugation of anti-CD4 monoclonal antibody to magnetic nanoparticles as a highly efficient affinity adsorbent for positive selection of peripheral blood T CD4+ lymphocytes. Int. J. Biol. Macromol. 161, 729–737. 10.1016/j.ijbiomac.2020.05.264 32497673

[B71] Reguera J. de Aberasturi D. J. Henriksen-Lacey M. Langer J. Espinosa A. Szczupak B. (2017). Janus plasmonic–magnetic gold–iron oxide nanoparticles as contrast agents for multimodal imaging. Nanoscale 9, 9467–9480. 10.1039/c7nr01406f 28660946

[B72] Robinson P. J. Dunnill P. Lilly M. D. (1973). The properties of magnetic supports in relation to immobilized enzyme reactors. Biotechnol. Bioeng. 15, 603–606. 10.1002/bit.260150318

[B73] Sang L. Li J. Zhang F. Jia J. Zhang J. Ding P. (2022). Glycyrrhetinic acid modified chlorambucil prodrug for hepatocellular carcinoma treatment based on DNA replication and tumor microenvironment. Colloids Surfaces B Biointerfaces 220, 112864. 10.1016/j.colsurfb.2022.112864 36272286

[B74] Sharifi M. Reza Avadi M. Attar F. Dashtestani F. Ghorchian H. Rezayat S. M. (2019). Cancer diagnosis using nanomaterials based electrochemical nanobiosensors. Biosens. Bioelectron. 126, 773–784. 10.1016/j.bios.2018.11.026 30554099

[B75] Shen Y. Wu C. Uyeda T.Q. P. Plaza G. R. Liu B. Han Yu (2017). Elongated nanoparticle aggregates in cancer cells for mechanical destruction with low frequency rotating magnetic field. Theranostics 7, 1735–1748. 10.7150/thno.18352 28529648 PMC5436524

[B76] Shen Z. Song J. Bryant C. Y. Zhou Z. Wu A. Chen X. (2018). Emerging strategies of cancer therapy based on ferroptosis. Adv. Mater. 30, 1704007. 10.1002/adma.201704007 PMC6377162 29356212

[B77] Short N. J. Rytting M. E. Cortes J. E. (2018). Acute myeloid leukaemia. Lancet 392, 593–606. 10.1016/S0140-6736(18)31041-9 30078459 PMC10230947

[B78] Shuvaev S. Akam E. Peter C. (2021). Molecular MR contrast agents. Investig. Radiol. 56, 20–34. 10.1097/RLI.0000000000000731 33074931 PMC7719082

[B79] Siegel R. L. Miller K. D. Jemal A. (2019). Cancer statistics, 2019. CA a cancer J. Clin. 69, 7–34. 10.3322/caac.21551 30620402

[B80] Sodipo B. K. Aziz A. A. (2016). Recent advances in synthesis and surface modification of superparamagnetic iron oxide nanoparticles with silica. J. Magnetism Magnetic Mater. 416, 275–291. 10.1016/j.jmmm.2016.05.019

[B81] Song C. Sun W. Xiao Y. Shi X. (2019). Ultrasmall iron oxide nanoparticles: Synthesis, surface modification, assembly, and biomedical applications. Drug Discov. today 24, 835–844. 10.1016/j.drudis.2019.01.001 30639557

[B82] Spirou S. V. Basini M. Lascialfari A. Sangregorio C. Innocenti C. (2018). Magnetic hyperthermia and radiation therapy: Radiobiological principles and current practice. Nanomaterials 8, 401. 10.3390/nano8060401 29865277 PMC6027353

[B83] Travis L. B. Andersson M. Gospodarowicz M. Van Leeuwen F. E. Bergfeldt K. Lynch C. F. (2000). Treatment-associated leukemia following testicular cancer. J. Natl. Cancer Inst. 92, 1165–1171. 10.1093/jnci/92.14.1165 10904090

[B84] Trujillo-Alonso V. Pratt E. C. Zong H. Lara-Martinez A. Kaittanis C. Rabie M. O. (2019). FDA-approved ferumoxytol displays anti-leukaemia efficacy against cells with low ferroportin levels. Nat. Nanotechnol. 14, 616–622. 10.1038/s41565-019-0406-1 30911166 PMC6554053

[B85] Walter R. B. (2022). Where do we stand with radioimmunotherapy for acute myeloid leukemia? Expert Opin. Biol. Ther. 22, 555–561. 10.1080/14712598.2022.2060735 35350938 PMC9090441

[B86] Wang J. Chen B. Cheng J. Cai X. Xia G. Liu R. (2011). Apoptotic mechanism of human leukemia K562/A02 cells induced by magnetic iron oxide nanoparticles co-loaded with daunorubicin and 5-bromotetrandrin. Int. J. nanomedicine 6, 1027–1034. 10.2147/IJN.S18023 21720514 PMC3124388

[B87] Wang L. Chen B. Lin M. Cao Y. Chen Y. Chen X. (2015). Decreased expression of nucleophosmin/B23 increases drug sensitivity of adriamycin-resistant Molt-4 leukemia cells through mdr-1 regulation and Akt/mTOR signaling. Immunobiology 220, 331–340. 10.1016/j.imbio.2014.10.015 25457413

[B88] Wang S. Luo J. Zhang Z. Dong D. Shen Y. Fang Y. (2018). Iron and magnetic: New research direction of the ferroptosis-based cancer therapy. Am. J. Cancer Res. 8, 1933–1946. 30416846 PMC6220147

[B89] Wei H. Wang E. (2013). Nanomaterials with enzyme-like characteristics (nanozymes): Next-generation artificial enzymes. Chem. Soc. Rev. 42, 6060–6093. 10.1039/c3cs35486e 23740388

[B90] Wen Q. Wang S. Yan J. Cong L. Chen Y. Xi H. (2014). Porous nitrogen-doped carbon nanosheet on graphene as metal-free catalyst for oxygen reduction reaction in air-cathode microbial fuel cells. Bioelectrochemistry 95, 23–28. 10.1016/j.bioelechem.2013.10.007 24239870

[B91] Wicki A. Witzigmann D. Balasubramanian V. Huwyler J. (2015). Nanomedicine in cancer therapy: Challenges, opportunities, and clinical applications. J. Control. release 200, 138–157. 10.1016/j.jconrel.2014.12.030 25545217

[B92] Winandy S. Wu P. Georgopoulos K. (1995). A dominant mutation in the Ikaros gene leads to rapid development of leukemia and lymphoma. Cell 83, 289–299. 10.1016/0092-8674(95)90170-1 7585946

[B93] Xie W. Guo Z. Gao F. Gao Q. Wang D. Liaw B. (2018). Shape-size-and structure-controlled synthesis and biocompatibility of iron oxide nanoparticles for magnetic theranostics. Theranostics 8, 3284–3307. 10.7150/thno.25220 29930730 PMC6010979

[B94] Yang L. Krefting I. Gorovets A. Marzella L. Kaiser J. Boucher R. (2012). Nephrogenic systemic fibrosis and class labeling of gadolinium-based contrast agents by the Food and Drug Administration. Radiology 265, 248–253. 10.1148/radiol.12112783 22923714

[B95] You N. Wang X.-F. Li J.-Y. Fan H.-T. Shen H. Zhang Q. (2019). Synergistic removal of arsanilic acid using adsorption and magnetic separation technique based on Fe3O4@ graphene nanocomposite. J. Industrial Eng. Chem. 70, 346–354. 10.1016/j.jiec.2018.10.035

[B96] Zhao Y. Chen X. Feng S. (2019). Autologous hematopoietic stem cell transplantation in acute myelogenous leukemia. Biol. Blood Marrow Transplant. 25, e285–e292. 10.1016/j.bbmt.2019.04.027 31054985

[B97] Zheng X. Fan X. Fu B. Zheng M. Zhang A. Zhong K. (2017). EpCAM inhibition sensitizes chemoresistant leukemia to immune surveillance. Cancer Res. 77, 482–493. 10.1158/0008-5472.CAN-16-0842 27697766

